# Retinal Nerve Fiber Layer Thickness Changes in Parkinson Disease: A Meta-Analysis

**DOI:** 10.1371/journal.pone.0085718

**Published:** 2014-01-21

**Authors:** Ji-guo Yu, Yi-fan Feng, Yi Xiang, Jin-hai Huang, Giacomo Savini, Vincenzo Parisi, Wan-ju Yang, Xun-an Fu

**Affiliations:** 1 Department of Ophthalmology, The Central Hospital of Wuhan, Hubei, China; 2 Department of Ophthalmology, Zhongshan Hospital, Fudan University, Shanghai, China; 3 The Affiliated Eye Hospital of Wenzhou Medical University, Zhejiang, China; 4 G.B. Bietti Eye Foundation-IRCCS, Rome, Italy; University of Ulm, Germany

## Abstract

**Background:**

Parkinson disease (PD) is a neurodegenerative process that leads to a selective loss of dopaminergic neurons, mainly in the basal ganglia of the brain. Numerous studies have analyzed the ability of optical coherence tomography (OCT) to detect retinal nerve fiber layer (RNFL) thickness abnormalities and changes in PD, but the results have not always been consistent. Therefore, we carried out a meta-analysis to evaluate the RNFL thickness measured with OCT in PD.

**Methods and Findings:**

Case-control studies were selected through an electronic search of the Cochrane Controlled Trials Register, PUBMED and EMBASE. For the continuous outcomes, we calculated the weighted mean difference (WMD) and 95% confidence interval (CI). The statistical analysis was performed by RevMan 5.0 software. Thirteen case-control studies were included in the present meta-analysis, containing a total of 644 eyes in PD patients and 604 eyes in healthy controls. The results of our study showed that there was a significant reduction in average RNFL thickness in patients with PD compared to healthy controls (WMD = −5.76, 95% CI: −8.99 to −2.53, *P* = 0.0005). Additionally, differences of RNFL thickness in superior quadrant (WMD = −4.44, 95% CI: −6.93 to −1.94, *P* = 0.0005), inferior quadrant (WMD = −7.56, 95% CI: −11.33 to −3.78, *P*<0.0001), nasal quadrant (WMD = −3.12, 95% CI: −5.63 to −0.61, *P* = 0.01) and temporal quadrant (WMD = −4.63, 95% CI: −7.20 to −2.06, *P* = 0.0004) were all significant between the two groups.

**Conclusion:**

In view of these results and the noninvasive nature of OCT technology, we surmise that OCT could be a useful tool for evaluating the progression of the Parkinson disease.

**Trial Registration:**

ClinicalTrials.gov NCT01928212

## Introduction

Optical coherence tomography (OCT) is a non-invasive retinal imaging technology that can provide high-resolution cross-sectional images of the peripapillary retinal nerve fiber layer (RNFL) and measure its thickness. Recently, a reduction of the RNFL thickness has been detected in several neurological disorders, such as multiple sclerosis [1], CADASIL [2], and Alzheimer's disease (AD) [3]. Different studies have reported RNFL changes also in Parkinson disease (PD) [4]–[7], a common neurodegenerative disease characterized by motor dysfunctions, originally described by James Parkinson in 1817 [8]. Previous studies on this subject, however, reported contradicting results. Some previous publications on PD did reported reductions of peripapillar RNFL thickness [5], [6], while others did not [7], [9]. In the present study, in order to determine whether RNFL thickness is reduced in PD patients, we performed a meta-analysis and systematically evaluated RNFL thickness measurements with OCT in a series of PD patients and in the healthy control groups.

## Materials and Methods

Following generally accepted methodology recommendations, this meta-analysis was performed according to the PRISMA (Preferred Reporting Items for Systematic Review and Meta-Analyses) statement (Checklist S1) [10]. The investigators wrote a protocol and registered it with the ClinicalTrials.gov Protocol Registration System (identification number: NCT01928212) in August 2013 [11].

### Search Strategy

The following electronic databases were searched: PubMed, Embase and the Cochrane Central Register of Controlled Trials up to 10 August 2013. A comprehensive search was conducted using the following terms “Parkinson disease”, “retinal nerve fiber layer”, “Optical coherence tomography”, “retinal thickness” and “RNFL”. Language restrictions were not used. Retrieved studies from both PubMed and Embase were imported into Refworks (version 1.0; Refworks, Bethesda, MD) where duplicate articles were manually deleted. Titles and abstracts of the remaining studies were independently scanned by 2 authors (J.G.Y. and Y.X). The full texts of the potentially relevant reports were then read to determine whether they met our inclusion criteria. In addition, the reference lists from all identified studies were also examined.

### Trials Selection

Studies fulfilling the following inclusion criteria were included in the present meta-analysis: (1) case-control studies; (2) patients with PD were compared with healthy controls; (3) all subjects underwent RNFL thickness measurement by OCT; (4) studies should provide the data of peripapillary RNFL thickness and (5) sample size ≥10 in each group. Exclusion criteria were as follows: (1) authors did not make RNFL measurements; (2) study without healthy control group; (3) the outcome values can not be used for meta-analysis and (4) duplicated articles. Two reviewers (J.G.Y and Y.F.F) separately evaluated studies based on inclusion and exclusion criteria, and discrepancies were resolved by discussion.

### Data Extraction

Using a standardized form, data from published studies were extracted independently by two reviewers (J.G.Y and J.H.H) to acquire the necessary information. From each of the included articles, the following information was retrieved: first author, publication time, country, OCT type, mean age, gender and number of eyes. The parapapillary RNFL thickness parameters evaluated in these studies were average thickness (360° measurement), temporal quadrant thickness (316–45°), superior quadrant thickness (46–135°), nasal quadrant thickness (136–225°) and inferior quadrant thickness (226–315°). Any discrepancies between the reviewers' results were resolved after discussion with another author (X.A.F).

### Quality Assessment

We assessed the methodologic quality of included studies based on the Newcastle-Ottawa Scale (NOS) for quality of case-control studies in meta-analysis [12], [13]. The NOS uses a star rating system to judge quality based on 3 dimensions of the study: selection, comparability, and exposure. The maximum for selection was 4 *, for comparability was 2 * and for outcome or exposure was 3 *. The maximum NOS score was 9 *, and the studies with ≥6 * were considered to have relatively higher quality. Based on these assessments, the two reviewers (J.G.Y. and Y.F.F.) independently evaluated the studies, and disagreements were resolved by discussion.

### Statistical Analysis

Original data were obtained from the articles as much as possible. Data that could not be obtained were to be calculated when necessary. When standard deviation (SD) was not available, it was calculated using the sample sizes and standard error. Statistical analysis was performed using RevMan software (version 5.0; Cochrane Collaboration, Oxford, United Kingdom). Summary estimates, including 95% confidence intervals (CIs), were calculated. For continuous outcome, means and standard deviations were used to calculate the weighted mean difference (WMD). The chi-square test, tau^2^ and the Higgins I^2^ test were used to assess heterogeneity [14]. The I^2^ test is a method for quantifying inconsistency across studies and describes the percentage of variability in effect estimates that is due to heterogeneity. A value greater than 50% is considered substantial heterogeneity. If there was no heterogeneity across studies (*P*>0.1, I^2^<50%), we adopt fixed-effects model for analysis. Otherwise random-effects model was used. Potential publication bias was examined using a funnel plot [15]. A *P* value less than 0.05 was considered statistically significant. Potential publication bias was examined using a funnel plot [16]. A strong correlation between sample size and summary estimates suggests publication bias.

## Results

### Study Selection

**Figure 1:**
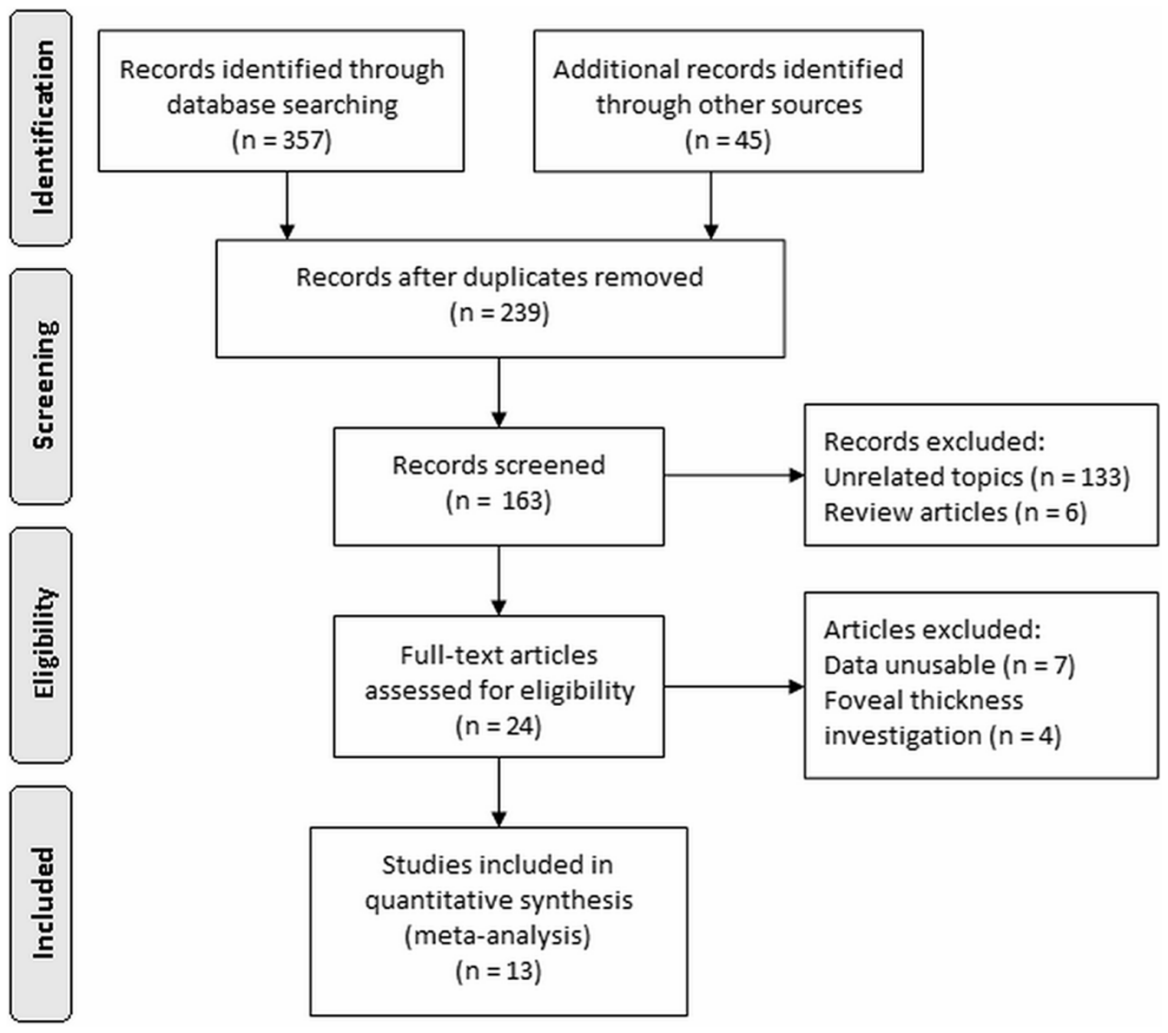
Search and study selection process.

The selection of studies is summarized in Figure 1. A total of 402 articles were initially identified; 239 records were excluded due to duplication, and 133 records were unrelated articles and 6 were review articles. 24 articles were left for further evaluation. However, of these 24 articles, 7 have not the useful data for analysis, and 4 were only investigating the macular fovea thickness, so they were not suitable for the inclusion criteria of meta-analysis. The left 13 case-control studies which met our inclusion criteria were included in the final meta-analysis [4]–[7], [9], [17]–[24].

### Characteristics and Quality of Studies

The studies were published between 2004 and 2013, and comprised a total of 1248 eyes (644 in the PD group and 604 in the control group). Two studies were done in Greece [5], [21], 3 in Turkey [6], [19], [23], 2 in Spain [17], [24], 1 each in Iran [20], Italy [22], USA [9], United Kingdom [7], Israel [18] and Germany [4]. The mean age of patients in most of studies ranged from 52 to 72 years and the percentage of female patients ranged from 42% to 90%. Sample sizes in these studies ranged from 20 to 200. The methodological qualities of included studies were assessed according to the NOS for assessing the case-control studies. Eleven of the 13 studies had scores ≥6 * and the mean score was 6.3. The characteristics and quality of these studies are showed in Table 1.

**Table 1:** Characteristics and quality of studies included in the Meta-analysis.

		OCT		No. of eyes		Mean Age (years)		Gender (Male/Female)		NOS Scale			
Author(s) Year	Country	Manufacturer	Model	PD	HC	PD	HC	PD	HC	Selection	Comparability	Expose	Total Score
Morgia/2013 [22]	Italy	Carl Zeiss	Stratus	81	86	65.6	65.5	24/19	34/52	***	*	**	6
Aaker/2010 [9]	USA	Heidelberg	Spectralis	18	19	64	67	13/5	9/10	****	*	**	7
Moschos/2011 [5]	Greece	Carl Zeiss	Stratus Model 3000	32	40	57	52	9/7	10/10	***	*	**	6
Albrecht/2012 [4]	Germany	Heidelberg	Spectralis	80	70	61.2	NR	30/10	NR	***	*	**	6
Tsironi/2012 [21]	Greece	Carl Zeiss	Stratus	24	24	66.6	64.3	14/10	11/13	***	**	**	7
Rohani/2012 [20]	Iran	Topcon	3D-OCT 1000 Mark II	54	50	54.6	55.0	40/14	34/16	****	*	**	7
Kirbas/2013 [19]	Turkey	Carl Zeiss	Cirrus HD	84	80	59.3	57.0	24/28	24/16	***	*	**	6
Inzelberg/2004 [18]	Israel	NR	NR	10	10	57	52	5/5	NR	***	*	**	6
Archibald/2011 [7]	UK	Carl Zeiss	Stratus Model 3000	34	17	71.6	71.3	22/15	9/10	****	**	**	8
Satue/2013 [17]	Spain	Carl Zeiss Heidelberg	Cirrus HD Spectralis	100	100	64	64	82/18	NR	**	*	**	5
Altintas/2008 [6]	Turkey	Carl Zeiss	Stratus Model 3000	34	22	59.3	58.1	9/8	6/5	**	*	**	5
Garcia-Martin/2012 [24]	Spain	Carl Zeiss Heidelberg	Cirrus HD Spectralis	75	75	64.4	64.2	45/30	45/30	***	*	**	6
Sen/2013 [23]	Turkey	Optovue	RTVue-100	18	11	63.56	61.18	8/10	5/6	****	**	**	8

PD = Parkinson's disease; HC = healthy controls; RNFL = retinal nerve fiber layer; NOS = Newcastle-Ottawa Scale; NR = not reported.

### Meta-analysis

**Figure 2:**
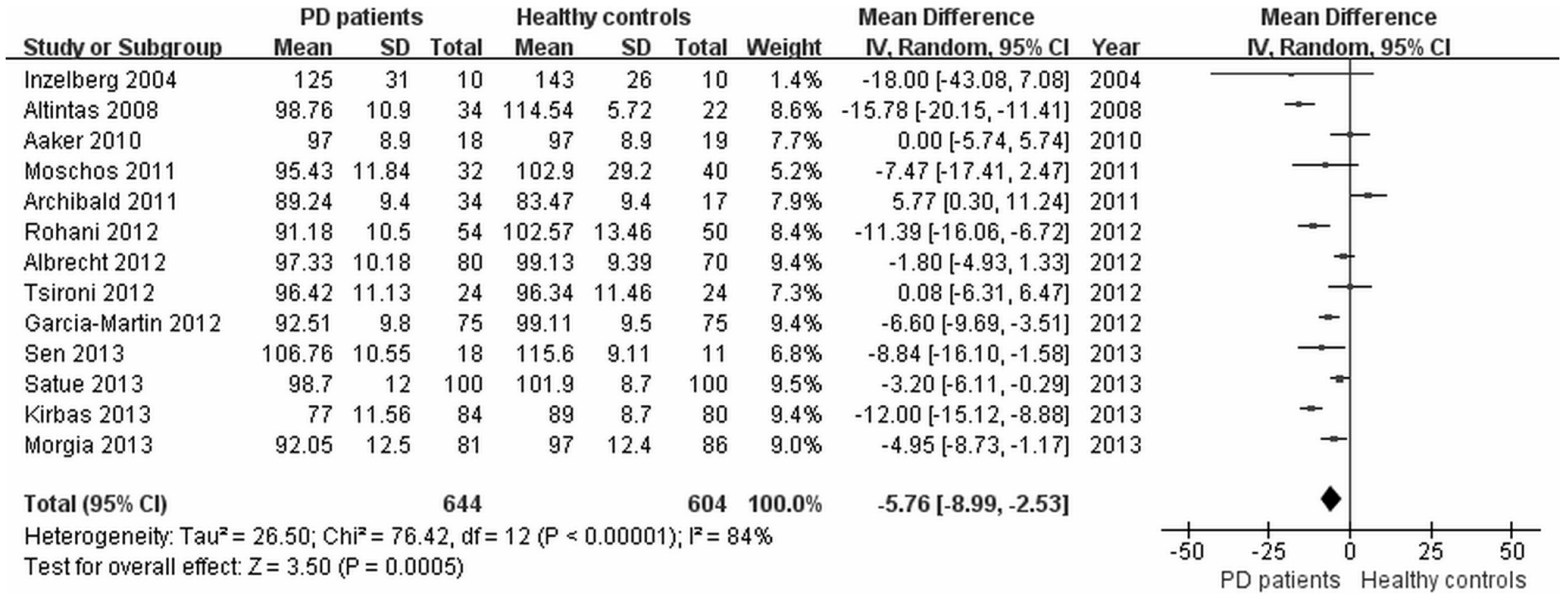
Meta-analysis of average RNFL thickness between PD patients and healthy controls.

**Figure 3:**
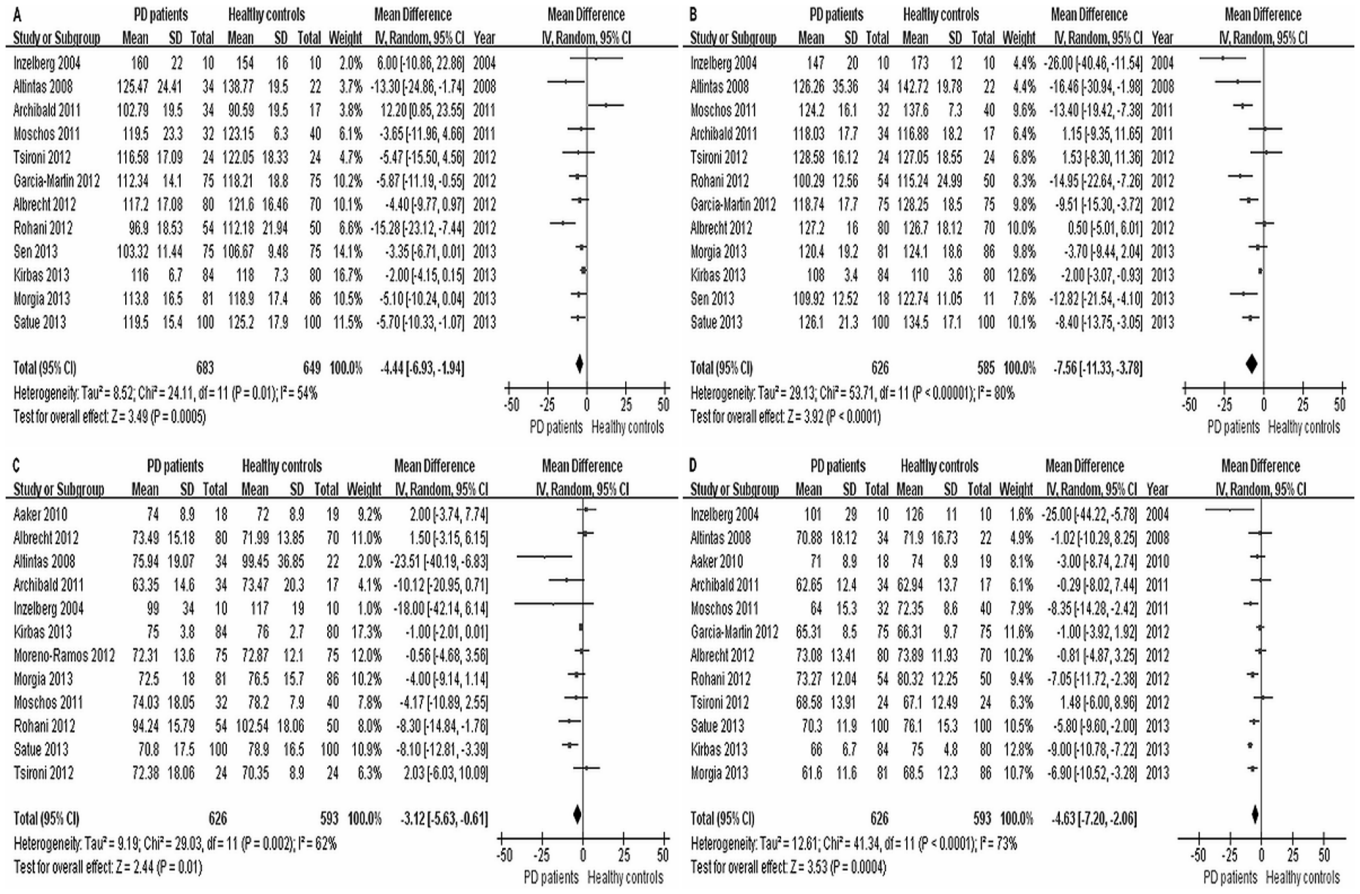
Meta-analysis of RNFL thickness between PD patients and healthy controls: (A) superior, (B) inferior, (C) nasal and (D) temporal.

Analysis of average RNFL thickness in 13 studies between PD patients and healthy controls found significant heterogeneity (*I^2^* = 84%) across the articles, so the data was pooled through the random effects model. The meta-analysis of these data showed that the average RNFL thickness in PD was reduced significantly compared with healthy controls (WMD = −5.76, 95% CI: −8.99 to −2.53, *P* = 0.0005, Figure 2). Moreover, RNFL thickness in each quadrant between PD patients and healthy controls were used for meta-analysis. The results showed that there was a significant difference of RNFL thickness between the two groups in superior quadrant (WMD = −4.44, 95% CI: −6.93 to −1.94, *P* = 0.0005, Figure 3A), inferior quadrant (WMD = −7.56, 95% CI: −11.33 to −3.78, *P*<0.0001, Figure 3B), nasal quadrant (WMD = −3.12, 95% CI: −5.63 to −0.61, *P* = 0.01, Figure 3C) and temporal quadrant (WMD = −4.63, 95% CI: −7.20 to −2.06, *P* = 0.0004, Figure 3D). In summary, the results of meta-analysis showed that there was a significant RNFL thickness reduction in all quadrants in PD patients compared with the control group.

### Subgroup Analysis

Of the 13 studies, 12 studies reported the types of OCT used, which included Stratus OCT (Carl Zeiss Meditec), Cirrus HD OCT (Carl Zeiss Meditec), Spectralis OCT (Heidelberg Engineering Inc.), 3D-OCT 1000 Mark II (Topcon Inc.) and RTVue-100 OCT (Optovue Inc.). Subgroup analyses were performed to examine whether differences in RNFL thickness were attributable to different types of OCT. The results showed that there were statistically significant differences of RNFL thickness in average and temporal quadrant in PD patients compared with the healthy controls regardless of types of OCT. However, for other three quadrants, no significant difference was found in certain kind of OCT. (Table 2).

**Table 2:** Subgroup analysis according to types of Optical Coherence Tomography.

	No. of studies	WMD (95% CI) of RNFL thickness	*P* Value
Stratus OCT	5		
average		−5.53 [−7.82, −3.24]	<0.00001
superior		−3.94 [−7.53, −0.34]	0.03
inferior		−6.51 [−9.99, −3.02]	0.0003
nasal		−4.38 [−7.76, −1.00]	0.01
temporal		−4.99 [−7.57, −2.42]	0.0001
Spectralis OCT	4		
average		−3.05 [−4.62, −1.47]	0.0002
superior		−5.31 [−7.87, −2.75]	<0.0001
inferior		−5.54 [−11.07, −0.02]	0.05
nasal		−3.19 [−8.64, 2.27]	0.25
temporal		−4.16 [−6.23, −2.10]	<0.0001
Cirrus HD OCT	3		
average		−8.37 [−11.75, −5.00]	<0.00001
superior		−5.15 [−9.52, −0.79]	0.02
inferior		−6.29 [−11.92, −0.67]	0.03
nasal		−0.91 [−1.85, 0.02]	0.06
temporal		−6.21 [−7.56, −4.87]	<0.00001
3D-OCT	1		
average		−11.39 [−16.06, −6.72]	<0.00001
superior		−15.28 [−23.12, −7.44]	0.0001
inferior		−14.95 [−22.64, −7.26]	0.0001
nasal		−8.30 [−14.84, −1.76]	0.01
temporal		−7.05 [−11.72, −2.38]	0.003
RTVue-100	1		
average		−8.84 [−16.10, −1.58]	0.02
superior		−3.35 [−11.05, 4.35]	0.39
inferior		−13.72 [−22.44, −5.00]	0.002

### Publication Bias

**Figure 4:**
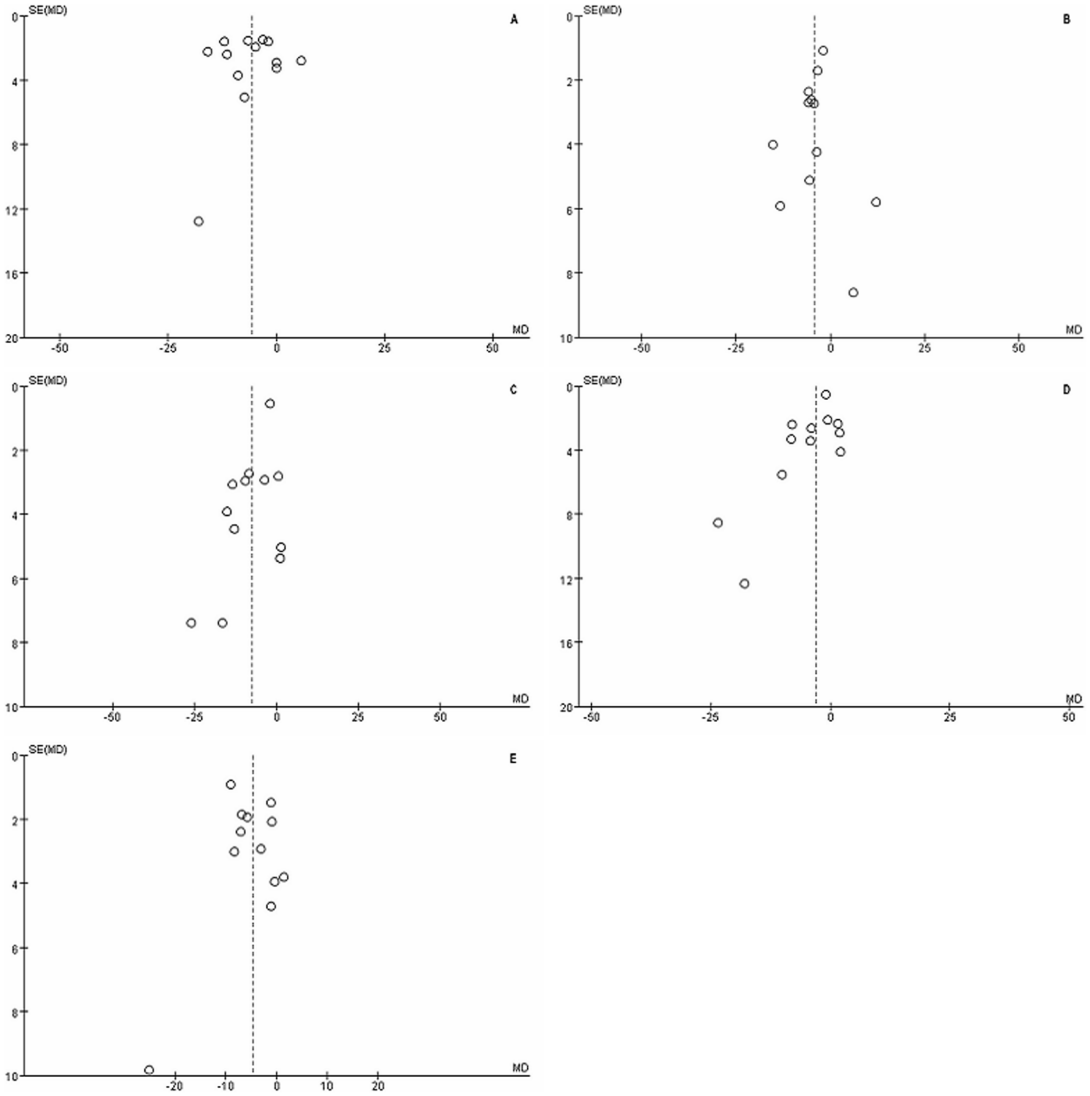
A funnel plot for evaluating the publication bias: (A) average, (B) superior, (C) inferior, (D) nasal and (E) temporal.

The funnel plot showed no correlation between study size and effect size or any other evidence of publication bias (Figure 4).

## Discussion

OCT is a fast, cost-efficient and non-invasive optical interferometric method generating cross-sectional images of the retina in vivo. OCT is a reliable noninvasive technique that enables quantitative assessment of the RNFL thickness around the optic nerve head and is considered as a most useful tool for the diagnosis and follow-up of neurodegenerative pathologies like optic neuritis [25], multiple sclerosis [26], migraine [27] and Alzheimer's disease [28], [29]. As evidenced by the reduction in the average RNFL thickness examined with high resolution OCT, OCT technology could be a viable biomarker for evaluating progressive thinning of RNFL over time in the neurodegenerative disorders.

PD is still a commonly encountered disease among aging population, but the accuracy of the clinical diagnosis of PD is still limited. Especially in the early stages, when cardinal symptoms are not conclusive, diagnosis can be delayed as structural neuroimaging methods such as CCT or MRI do not provide characteristic features that allow the diagnosis of this chronic neurodegenerative disorder [30]. For example, it is not always easy to distinguish it from essential tremor, the another neurological disorder typically involving a tremor of the arms, hands or fingers but sometimes involving the head or other body parts during voluntary movements such as eating and writing [31]. Given the value of RNFL examination as a method of detecting neurodegenerative disease progression and facilitating diagnosis of diseases, the aim of this meta-analysis was to evaluate the RNFL thickness changes in patients with PD.

Basis on the published data from 13 articles, our meta-analysis showed a significant reduction in average circumpapillary RNFL in PD patients comparing with the healthy controls. Moreover, further analysis provided evidence that there were significant differences of RNFL thickness between the two groups in superior, inferior, nasal and temporal quadrants. However, the different types of OCT may give different results. However, our subgroup analyses showed that the significant differences of RNFL thickness were found only in average and temporal quadrant by using all types of OCT. For other three quadrants, no significant difference was found in certain kind of OCT. One explanation may be that temporal fibers are characteristically susceptible in neurodegenerative diseases [32]. Previous studies reported a preferential loss of RNF in the temporal quadrants in PD, consistent with the involvement of the papillomacular bundle [5], [18], [33]. Another explanation for the results of subgroup analyses may be attributed to the small sample size, different PD severity and disease duration [17]. Although the differences of RNFL thickness in superior, inferior or nasal were not significant between the PD and healthy control groups examined by some types of OCT, the tendency of reductions in PD group were obvious.

Visual deficits are common in PD and include abnormal contrast sensitivity, motion perception abnormalities, impaired visual acuity and color vision, and visual hallucinations [34]. However, the exact loci of this impairment remain unclear. It is reported that the visual impairment in PD is due to dopaminergic loss in the retina [35]. Dopamine (DA) is important in controlling the efficiency of some neurochemical systems such as glutamate, γ-aminobutyric acid and glycine in the retina. Retinal DA deficiency alters visual processing by modification of receptive-field properties of ganglion cells [36]. Dysfunctions that result from DA depletion may involve long-term complex synaptic effects. It is possible that impoverished dopaminergic input to a subset of ganglion cells, contributes to abnormal production of glutamate and atrophy of these selected fibers [18]. Moschos et al [5] correlated multifocal ERG (mfERG) in the central retina in PD with OCT thinning of the NFL. Such morphological/functional correlation is reassuring. Further correlative studies of functional vision such as contrast sensitivity, color vision and the pattern ERG could be revealing.

The aim of this meta-analysis was to evaluate the usefulness of RNFL measurements as biomarkers of PD. However, there is growing evidence that the loss of DA amacrine cells may not be solely responsible for inner retinal thinning, because dopaminergic (DA) neurons and their interconnecting plexus are below the ganglion cells [37] and the processes of DA amacrine cells do not directly synapse onto ganglion cells. In addition, previous studies reported levodopa therapy did not completely restore the ERG in PD [38]. Recently, a spectral-domain OCT (SD-OCT) study provided direct evidence of the thinning of the pre-NFL of the retina in PD. In this study, Spund et al. reported that foveal pit was thinner and broader in PD and the difference became evident in an annular zone between 0.5 and 2 mm from the foveola, which nearly devoid of NFL [38]. More studies evaluation of macular thickness and volume by OCT in PD patients has yet to be done.

Moreover, glaucoma needs to be considered in the differential diagnosis of retinal NFL thinning. Glaucoma is a disease of the elderly and is often discovered on careful eye examination only, as early on many patients do not complain of any visual loss. Patients with AD and PD may have an increased occurrence rate of glaucoma. According to some studies, the incidence of glaucoma is about 23% among PD patients [39] compared to its incidence of 5–12% in the healthy, same age group [40]. In the current meta-analysis, all studies have reported that the participants with glaucoma were excluded basing on some of the following: classical appearance of the open angle glaucoma changes in the optic disc, enlargement and especially vertical enlargement of the optic cup, localized loss of disc rim and rim pallor, asymmetry of cup/disc ratio >0.2 between the two eyes, baring of the lamina cribrosa, intraocular pressure and typical visual field loss.

This meta-analysis may have some limitations. First, our review is restricted to studies published in indexed journals or in certain trial registers and conference proceedings. We did not search for unpublished studies or original data. Second, RNFL thickness is correlated with the PD severity, disease duration and physiologic aging [17]. Unfortunately, we did not make subgroup analysis for this due to lack of data. Last, studies included in our meta-analysis examined patients with different types of OCT. Different OCT manufacturers provide different ‘canned’ measures and manual measurements are not standardised either across equipments or investigators [41]. Further studies are needed to evaluate the specific retinal area of interest to study and the sensitivity and specificity of OCT in PD.

In conclusion, the results of meta-analysis showed that RNFL thickness decreased in all quadrants in PD patients compared with the healthy control group. In view of these results and the noninvasive nature of OCT technology, we believe that OCT could be a useful tool for evaluating the progression of the neurodegenerative disorders such as PD.

## Supporting Information

Checklist S1 (DOC)
